# Gap junction-mediated transfer of miR-145-5p from microvascular endothelial cells to colon cancer cells inhibits angiogenesis

**DOI:** 10.18632/oncotarget.8583

**Published:** 2016-04-05

**Authors:** Dominique Thuringer, Gaetan Jego, Kevin Berthenet, Arlette Hammann, Eric Solary, Carmen Garrido

**Affiliations:** ^1^ INSERM, U866, Faculty of Medecine, 21000 Dijon, France; ^2^ University of Bourgogne-Franche-Comté, 21000 Dijon, France; ^3^ INSERM, U1170, Institut Gustave Roussy, 94508 Villejuif, France; ^4^ CGFL, BP77980, 21000 Dijon, France

**Keywords:** GJIC, Cx43, micro-RNA, tubulogenesis, colorectal tumor

## Abstract

Gap junctional communication between cancer cells and blood capillary cells is crucial to tumor growth and invasion. Gap junctions may transfer microRNAs (miRs) among cells. Here, we explore the impact of such a transfer in co-culture assays, using the antitumor miR-145 as an example. The SW480 colon carcinoma cells form functional gap junction composed of connexin-43 (Cx43) with human microvascular endothelial cells (HMEC). When HMEC are loaded with miR-145-5p mimics, the miR-145 level drastically increases in SW480. The functional inhibition of gap junctions, using either a gap channel blocker or siRNA targeting Cx43, prevents this increase. The transfer of miR-145 also occurs from SW480 to HMEC but not in non-contact co-cultures, excluding the involvement of soluble exosomes. The miR-145 transfer to SW480 up-regulates their Cx43 expression and inhibits their ability to promote angiogenesis. Our results indicate that the gap junctional communication can inhibit tumor growth by transferring miRs from one endothelial cell to neighboring tumor cells. This “bystander” effect could find application in cancer therapy.

## INTRODUCTION

Gap junctions are specific cell-to-cell channels formed by membrane proteins called connexins (Cx). Among the Cx family, Cx43 is frequently down-regulated in human tumors, *e.g.* its loss is associated with cancer progression [1–5]. Gap channels permit the direct exchange of small molecules less than 1.5 kDa, between cells. Gap channels mainly composed of Cx43, are also permeable to micro-RNAs (miRs) with diameters of about 1.0 nm, and can transfer miRs from one cell to neighboring cells [6–10].

MiRs are endogenously processed non-coding RNAs that regulate gene expression at the transcriptional level [11]. MiRs also function as intercellular signals mediated by exosomes [12–15] or gap junctions [6–9, 16–18]. MiRs act as tumor suppressor or oncogene, depending on the receiver cells [19–23].

We have shown that the SW480 colon carcinoma cell line formed functional gap junction composed of Cx43 with microvascular endothelial cells (HMEC) [5]. Here, we explore the ability of gap junctions to drive miRs exchange between endothelial and tumor cells. MiR-145, which is downregulated in early stage of colorectal cancer [21, 24] and acts as tumor suppressor [25–27], is used as an example. In co-culture assays, we demonstrate that miR-145 can be transferred through gap junction channels, from endothelium to adjacent cancer cells, and vice-versa, and functions as an “antiangiogenic” signal. The “bystander” effects of gap junctions provide a new guiding strategy for the clinical application of miRs in cancer therapy.

## RESULTS

### Gap junctions mediate miR-145 transfer from endothelial to colon cancer cells

**Figure 1:**
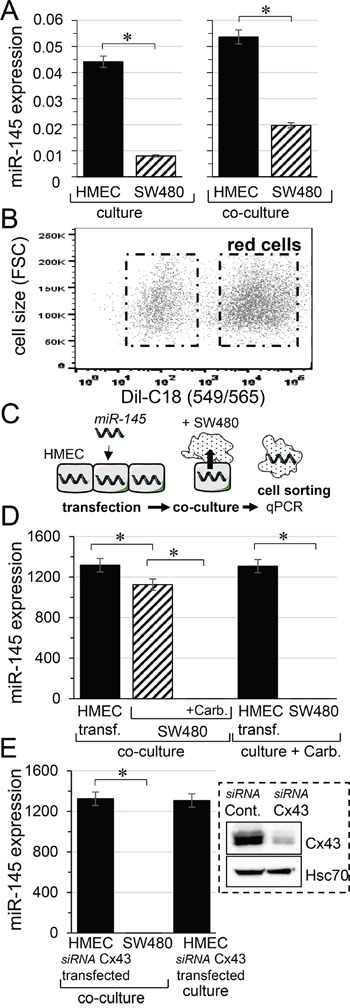
Micro-RNA transfer from microvascular endothelium (HMEC) to colorectal cancer cells (SW480) **A.** Expression profile of miR-145 in HMEC (black) and SW480 (hatched), separately cultured (left) or co-cultured (right) for 12 hours. Levels of miR-145 were expressed relative to levels of U6 snRNA, commonly used as an internal control in miRs analysis (means ± SD; *P<0.05; *n* = 3). **B.** Cell sorting by flow cytometry. SW480 were labelled with the fluorescent dye DiL-C18 (red cells), then plated with unlabeled HMEC in a ratio of 1:1. **C.** Scheme illustrating the procedure to determine the transfer of microRNA (miR-145) through hetero-cellular gap junction channels established between HMEC and SW480. **D.** Transfer of miR-145 to SW480 is inhibited by a gap junction blocker. HMEC loaded with miR-145-5p mimic (30 nM) were co-cultured with SW480, in the presence or the absence of carbenoxolone (carb. 100 μM). The miR-145 levels measured in donor HMEC (black) and receiver SW480 (hatched), after 12 h of co-culture. Note that carbenoxolone does not affect the miR-145 expression in transfected HMEC or in cancer cells cultured separately. **E.** Down-regulation of Cx43 expression in HMEC does not affect loading of miR-145 mimic but suppresses transfer of miR-145 to SW480. Right insert, representative immune-blot of Cx43 protein level in HMEC transfected with control siRNA or siRNA Cx43 for 2 days (n=5; Hsc70 as loading control). Note that the siRNA Cx43 transfection of HMEC does not affect their loading with miR-145-5p mimic. D, E. Values of miR-145-5p expression relative to U6 snRNA in each cell type and condition, are means ± SD of triplicate measurements from three experiments; *P<0.5 *vs* donors (Mann-Whitney U test and Kruskal-Wallis test; *n* = 3).

We first determined the basal level of miR-145-5p in HMEC and SW480 cells, cultured separately for 12 hours. We observed that miR-145-5p level was lower in SW480 than in HMEC (Figure 1A, left panel). Then, SW480 were labelled with the cell tracker DiL-C18 [28], and the two cell types were co-cultured for 12 hours before flow cytometry sorting (Figure 1B). The mir145-5p levels increased by 20% in HMEC and by 60% in SW480 cells after co-culture (Figure 1A, right panel). To determine whether miR-145 is transferred from endothelial to cancer cells, we transfected HMEC with miR-145-5p mimic (30 nM) then we cultured them with DiL-C18-labelled SW480 (ratio 1:1). Such a transfection did not affect the adhesion of SW480 to HMEC. After 12 hours of co-culture, the cell types were sorted by flow cytometry and miR145-5p level was determined in each population (Figure 1C). Both HMEC and SW480 expressed high levels of miR-145-5p (Figure 1D). To evaluate the contribution of gap junctions to the miR-145 transfer into SW480, co-culture was made in the presence of carbenoxolone, known to block the gap junction intercellular communication (GJIC) [5, 8]. Clearly, inhibition of GJIC prevented the increase in miR-145-5p in SW480 (Figure 1D). It should be noted that the relative value measured in these conditions was similar to that measured in cells cultured alone (*i.e.* 0.00761±0.0004 with carbenoxolone compared to 0.00801±0.0004 in SW480 alone, n=3; P>0.5; see Figure 1A; left panel). Because Cx43 is mostly involved in GJIC between HMEC and SW480 [5], we performed the same experiment after knocking down the Cx43 expression in HMEC by using small-interfering RNA (insert, Figure 1D). The down-regulation of Cx43 in HMEC prevented the transfer of miR-145-5p to SW480 within 12 hours of co-culture (Figure 1E). The miR-145 expression level measured in SW480 in these conditions (0.00728±0.0005; n=3) was similar to that measured in SW480 cultured alone (0.00801±0.0004; n=3; P>0.5).

### The miR-145 transfer between HMEC and SW480 is bi-directional

**Figure 2:**
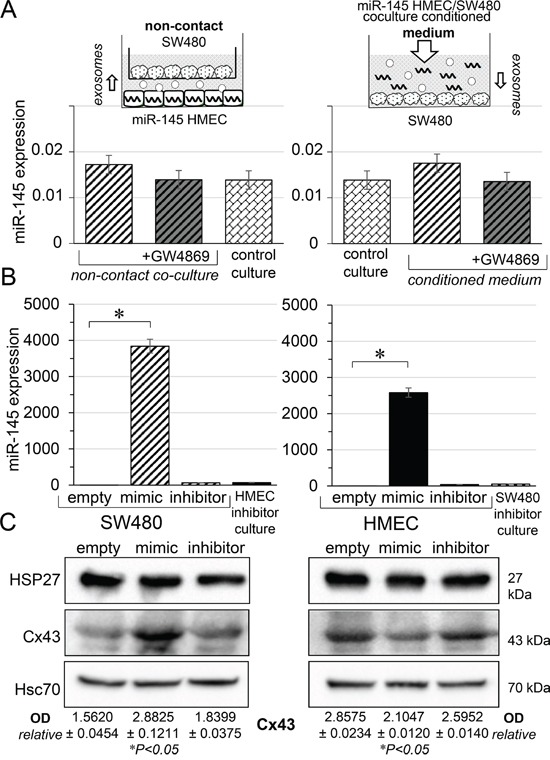
Gap junction channels, not exosomes, mediated miR-145 transfer between cells **A.** Scheme illustrating the procedure to evaluate the contribution of paracrine pathways to the intercellular miR transfer. Left panels: Receiver SW480 were co-cultured with miR-145-loaded HMEC with no physical contact (non-contact). Right panels: Receiver SW480 were exposed to the cell conditioned medium collected from direct co-cultures of miR-145-loaded HMEC / SW480 for 7 hours (medium). Control cultures are untreated homotypic cultures. HMEC loaded with miR-145-5p mimic (60 nM). Levels of miR-145 expression relative to U6 snRNA were measured in SW480 after 7 hours of cell incubation. No significant decrease was induced by the exosome inhibitor, GW4869 (10 μM). Means ± SD; *P*>0.05 vs control culture. **B.** miR-145 expression in receiver cells, SW480 (left panel) and HMEC (right panel), after co-culture for 7 hours with donor cells, HMEC and SW480, respectively. Cell donors were loaded or not with miR-145-mimic (60 nM) or miR-145-inhibitor (60 nM). Hatched area, receiver SW480; black area, receiver HMEC (U6 snRNA as internal control; mean ± SD; *P<0.01 *vs* empty; Mann-Whitney U test and Kruskal-Wallis test; *n* = 3). Note that values measured by qPCR are biased in cell loaded with miR inhibitor due to the sequence similarity with miR-145-5p. **C**. Cell expression of Cx43 and HSP27 in receiver cells, SW480 (left panel) and HMEC (right panel), co-cultured for 7h in the same conditions as in **B**. Immunoblots representative of 5 experiments (Hsc70 as loading control; mean ± SD; *P<0.5 *vs* empty; Mann-Whitney U test and Kruskal-Wallis test; *n* = 3).

We have previously shown that functional gap junction are established between HMEC and SW480 within a few hours of co-culture [5]. Accordingly, in co-culture experiments reported in Figure 1, 7 hours were sufficient to observe miR-145-5p cell transfer (not shown). Therefore, in the following experiments, we collected and analyzed cell contains after 7 hours of culture or co-culture. We first cultured SW480 with miR-145-5p-transfected HMEC in transwell plates to prevent any cell-cell contact. Secondly, SW480 were cultured in the cell conditioned medium collected from direct co-cultures of transfected HMEC and SW480 (Figure 2A, upper panels). In both conditions, we failed to detect any increase in miR-145-5p level in SW480 (Figure 2A, lower panels). These experiments were repeated in the presence of the exosome release inhibitor, GW4869 (10 μM) [15], which did not significantly modify the low level of miR-145-5p in SW480. These results indicate that SW480 do not ingest extracellular miR-145-5p, either free or incorporated into soluble exosomes. In contrast, a high level of miR-145 was measured in SW480 co-cultured with transfected HMEC for 7 hours (left side; Figure 2B). To test the selectivity of the GJIC, we transfected either SW480 or HMEC with the same dose of miR-145-5p mimic or miR-145-5p inhibitor (60 nM). Transfected SW480 were co-cultured with non-transfected HMEC, and vice-versa. A high level of miR-145 was measured in non-transfected cells co-cultured with mimic-transfected cells, but not with the inhibitor (Figure 2B). The miR-145-5p transfer appeared to be slightly more efficient from HMEC to SW480 than the other way around (*i.e.* after co-culture, miR-145 level was 3,840±192 in non-transfected SW480 co-cultured with transfected HMEC, and 2,580±129 in non-transfected HMEC co-cultured with transfected SW480; *P*<0.05, n=3). Immunoblot analyses identified approximately a 2-fold increase in Cx43 expression in non-transfected SW480 after 7 h of co-culture with miR-145-5p transfected HMEC cells. In contrast, a 20% decrease in Cx43 expression was observed in non-transfected HMEC cells co-cultured with miR-145-5p transfected SW480 cells (Figure 2C). These effects were not observed with the miR-145-5p inhibitor. Of note, the expression of the small heat shock protein HSP27, which promotes the establishment of GJIC between endothelial and cancer cells [5], remained unchanged in all conditions (Figure 2C).

### The transfer of miR-145-5p to cancer cells inhibits their proangiogenic effect *in vitro*

**Figure 3:**
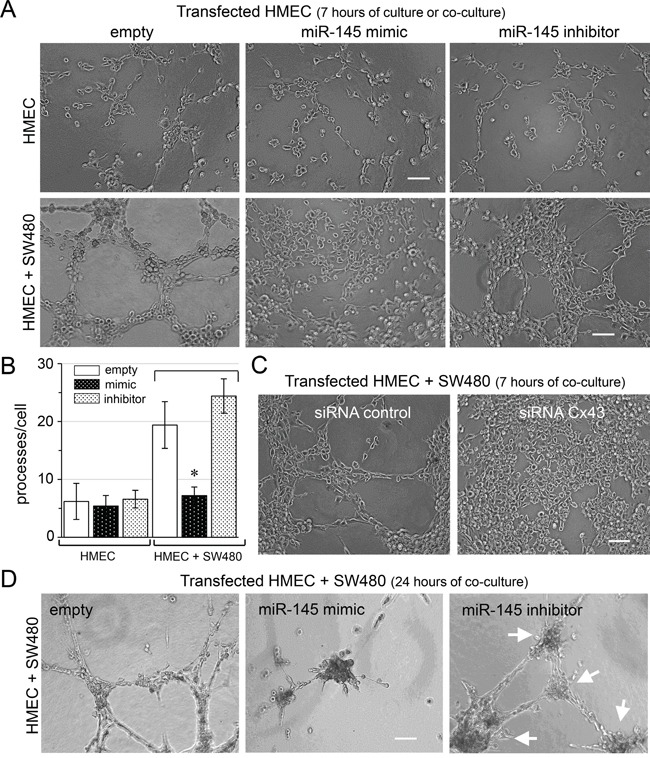
Antiangiogenic effect of miR-145 transfer *in vitro* *In vitro* tubulogenesis assay of HMEC loaded or not (empty) with miR-145-5p mimic (60 nM) or inhibitor (60 nM). Donor HMEC were plated on Matrigel, and incubated or not with SW480 for 7 h or 24 h, as indicated. **A, C, D.** Representative micro-photographs of endothelial tube formation. Note the adhesion of SW480 onto the endothelial neo-tubes (Bar 80 μm; n=3 experiments triplicate). **A.** Antiangiogenic effects of miR-145. **B.** Histogram shows the number of branch points per field of view (at least 80 single cells were scored). HMEC cultured for 7h as in **A** (mean ± SD; *P<0.01 *vs* empty; Mann-Whitney U test and Kruskal-Wallis test; *n* = 3). **C.** Antiangiogenic effects of siRNA Cx43 knockdown in HMEC. **D.** Tumor growth development induced by miR-145 inhibitor, after 24 hours of co-culture. Arrows indicated tumor development. Representative of three experiments with similar results.

We subsequently used an *in vitro* matrigel tube formation assay to explore if miR-145-5p could modulate the formation of capillary-like structures by HMECs [29]. Transfection of miR-145-5p mimic did not affect the ability of HMEC to form these structures when plated alone in homotypic cultures for 7 hours (Figure 3A). Importantly, co-culture of empty-transfected HMEC and SW480 increased the formation of typical capillary-like structures within 7 hours (Figure 3A, lower panel, and Supplementary Figure S1). When miR-145-5p mimic-transfected HMEC were co-cultured with non-transfected SW480, the formation of a capillary-like network was inhibited, suggesting an anti-angiogenic effect of miR-145-5p. Such an effect was not observed when HMEC were transfected with its specific inhibitor (Figure 3A, lower panels and Supplementary Figure S1). Thus miR-145-5p acts as a potent anti-angiogenic factor, inhibiting the tubulogenesis induced by cancer cells (Figure 3B). The same observation was made when miR-145-5p mimic-transfected SW480 were co-cultured with HMEC (not shown). Importantly, inhibition of gap junctional transfer from HMEC to SW480 through the down-regulation of Cx43 expression in HMEC improved endothelial tube formation in the presence of SW480, suggesting that expression of miR-145-5p in SW480 was required to inhibit the proangiogenic effect of the co-culture (Figure 3C). Importantly, the transfer of miR-145-5p from endothelial to colon cancer cells could decrease tumor growth along the capillary-like network, which can be reversed by the miR-145-5p inhibitor (Figure 3D). Taken together, these experiments suggest that the transfer of miR-145 from HMEC to SW480 could down-regulate colon cancer cell growth by preventing the formation of new vessels.

## DISCUSSION

We previously demonstrated that SW480 cells derived from a primary colorectal tumor secrete high levels of HSP27 that promotes the formation of gap junction between tumor and endothelial cells [5]. Here, we show that these gap junction channels permit the passage of miR-145-5p from endothelial cells to cancer cells which in turn up-regulates Cx43 expression in cancer cells and inhibits their proangiogenic effect.

That gap junction channels formed by Cx43 mediate the transfer of miR-145 from endothelial to cancer cells, which is demonstrated here by: (1) exosomes or other secreted structures are not involved as this transfer is prevented by the absence of cell-to-cell contact; (2) the exosome inhibitor, GW4869, does not reduce the transfer of miR-145 from HMEC to SW480, as previously described for its transfer from smooth muscle cells to endothelial cells [23]; (3) the functional inhibition of gap junctions using either a chemical blocker or siRNA mediating Cx43 down-regulation, prevents miR-145 transfer from HMEC to SW480; and (4) miR-145 transfer is also observed from cancer cells to endothelial cells. We conclude that gap junction channels are the main route for the passage of miR-145 between adjacent cells in blood capillaries. Our results corroborate previous reports showing that various miRs are transferred through gap junctions formed by Cx43 between normal and cancer cells [8, 9, 18, 23].

The miR-145 transfer from endothelial to cancer cells prevents vessel tube formation *in vitro*. This is in agreement with previous reports showing the inhibitory effect of miR-145 on angiogenesis and, consequently, tumor growth [19, 22, 30]. As tumor cells grow, the formation of new immature blood vessels increases together with the demand for nutrients. It can be speculated that to maintain the proangiogenic phenotype of endothelial cells, the level of miR-145 in the surrounding tissue has to be low. This is the case of the early step in colon cancer progression where miR-145 expression is usually down-regulated [21, 25, 26, 31]. Indeed primary tumors have decreased levels of miR-145 compared with normal samples of the same tissue. Thus it is possible that downregulation of miR-145 is associated with an increased angiogenesis in tumors because the stabilizing effect on endothelium is decreased or lost [23].

The loss of gap junctions is involved in the carcinogenesis process [1–5]. GJIC is inhibited by most of the tumor promoters [32, 33]. The restoration of GJIC, by transfection of cDNAs encoding Cx43 proteins, inhibits the aberrant growth rates of tumor cells [34, 35]. We report here that miR-145 up-regulates the expression of Cx43 in SW480. Such an effect was also observed in the normal corneal epithelium thereby modulating tissue stabilization [36]. We do not have identified the precise genes or signals involved here, because of the enormous complexity of posttranscriptional regulation of Cx gene expression [37, 38]. To date, a few miRs affecting Cx43 are known. Both miR-1 and miR-206 block Cx43 mRNA translation, downregulating Cx43 expression in muscles [39]. MiR-130a would act as a direct inhibitor of Cx43 in cardiac arrhythmias [40] as does miR-221/222 and miR-125b in human glioblastoma [41, 42]. In contrast to these oncogenic miRs, miR-145 increases Cx43 expression. It remains to be demonstrated if a common regulatory mechanism accounts for the miRs-induced changes in Cx43 expression in cancer cells.

The dysregulation of miRs expression, *i.e.* with an early down-regulation of miR-145, in colorectal cancer biopsy is associated with a worse tumor grading. Our data provide new insight into the transfer machinery of miRs from cell-to-cell. According to the “bystander effect”, specific miRs could transfer from a small number of cells to a larger number of neighboring cancer cells. This mechanism could be used to prevent tumor progression and/or increase the efficiency of cancer therapy [38, 43, 44].

## MATERIALS AND METHODS

### Cells

Human microvascular endothelial cells (HMEC; Lonza; Basel, Switzerland) were grown in DMEM plus 10% FCS (5% CO_2_; 37°C). Human colorectal cancer cell line, SW480 (ATCC CCL-228), was plated in DMEM plus 10% FCS. Cells were incubated overnight in FCS-free media before use.

### Reagents

Rabbit polyclonal anti-HSP27 was purchased from ABR (AffinityBioReagent, ThermoFisher, Fr). Rabbit polyclonal anti-Cx43 (C-20) and mouse anti-Hsc70 were from Santa Cruz Biotech. Vybrant cell labeling solution DiL-C18 was from Molecular Probes (Invitrogen). GW4869 was purchased from Calbiochem (Merck Chimie SAS, Fontenay-sous-Bois, Fr). Other chemicals were from Sigma-Aldrich.

### Transfection

Human hsa-miR-145-5p mimics (mirVana TM miRNA mimic, 4464066-MC11480) and hsa-miR-145-5p inhibitors (mirVana TM miRNA mimic, 4464084-MH11480) were purchased from Ambion (Invitrogen; Life Technologies, Saint-Aubin, Fr). Silencing RNA (siRNA) targeting the human Cx43 gene was purchased from Santa Cruz Biotech (GJA1_human mapping 6q22.31; Clinisciences; Nanterre, Fr) and control siRNA was from Dharmacon (ThermoFischer, Saint-Remy-les-Chevreuses, Fr). Cells were transfected by lipofectamine RNAiMAX according to the manufacturer's protocol (Invitrogen; Life Technologies).

### Co-culture and cell sorting by flow cytometry

Receiver cells were labelled with DiL-C18, then washed and mixed with unlabeled cells (donors) in a ratio of 1:1. After co-culture, donors and receivers were separated by flow cytometry based on the fluorescence dye. Cell sorts were carried out twice to guarantee 100% purity.

### RNA isolation and real-time PCR analysis

Total RNA was isolated using TRIzol reagent (Invitrogen). Expression of miR-145 was determined using TaqMan miRNA assay (Invitrogen) according the manufacturer's protocols. Level of miR-145 was expressed relative to the level of U6 snRNA (Ambion, 4427975-001973), used as internal control for each measurement. Relative values thus obtained are averaged.

### Immunoprecipitation

Briefly, cells were lysed in RIPA buffer, and immunoprecipitation was performed with antibodies, as previously described [29].

### Endothelial tube formation assay in collagen gels

HMEC were trypsinized and resuspended in ECM gel with DMEM according to the manufacturer's instructions (Cell Biolabs, Inc) [28]. Each well is duplicated for each experiment, and each experiment was repeated three times. For short term assays (after 7 hours of incubation at 37°C), 80 single cells were scored for the number of processes per cell. Cells were photographed at a magnification of x10 using Zeiss microscope, equipped with a video camera.

### Statistical analysis

Results are expressed as mean ± SD. Groups were compared using one-way analysis of variance (ANOVA; Statview Software). A Mann-Whitney *U* test was also used to compare data groups. In some cases, statistics were made with Tanagra software using a Kruskal-Wallis 1-way ANOVA. In all cases, *P values < 0.05 were significant.

## SUPPLEMENTARY FIGURE



## References

[1] Loewenstein WR, Kanno Y (1966). Intercellular communication and the control of tissue growth: lack of communication between cancer cells. Nature.

[2] Sirnes S, Lind GE, Bruun J, Fykerud TA, Mesnil M, Lothe RA, Rivedal E, Kolberg M, Leithe E (2015). Connexins in colorectal cancer pathogenesis. Int J Cancer.

[3] Yamasaki H, Mesnil M, Omori Y, Mironov N, Krutovskikh V (1995). Intercellular communication and carcinogenesis. Mutat Res.

[4] Bigelow K, Nguyen TA (2014). Increase of gap junction activities in SW480 human colorectal cancer cells. BMC Cancer.

[5] Thuringer D, Berthenet K, Cronier L, Solary E, Garrido C (2015). Primary tumor- and metastasis-derived colon cancer cells differently modulate connexin expression and function in human capillary endothelial cells. Oncotarget.

[6] Valiunas V, Polosina YY, Miller H, Potapova IA, Valiuniene L, Doronin S, Mathias RT, Robinson RB, Rosen MR, Cohen IS, Brink PR (2005). Connexin-specific cell-to-cell transfer of short interfering RNA by gap junctions. J Physiol.

[7] Valiunas V, Wang HZ, Li L, Gordon C, Valiuniene L, Cohen IS, Brink PR (2015). A comparison of two cellular delivery mechanisms for small interfering RNA. Physiol Rep.

[8] Katakowski M, Buller B, Wang X, Rogers T, Chopp M (2010). Functional microRNA is transferred between glioma cells. Cancer Res.

[9] Hong X, Sin WC, Harris AL, Naus CC (2015). Gap junctions modulate glioma invasion by direct transfer of microRNA. Oncotarget.

[10] Inose H, Ochi H, Kimura A, Fujita K, Xu R, Sato S, Iwasaki M, Sunamura S, Takeuchi Y, Fukumoto S, Saito K, Nakamura T, Siomi H, Ito H, Arai Y, Shinomiya K (2009). A microRNA regulatory mechanism of osteoblast differentiation. Proc Natl Acad Sci U S A.

[11] Visone R, Croce CM (2009). MiRNAs and cancer. Am J Pathol.

[12] Chen X, Liang H, Zhang J, Zen K, Zhang CY (2012). Secreted microRNAs: a new form of intercellular communication. Trends Cell Biol.

[13] Salido-Guadarrama I, Romero-Cordoba S, Peralta-Zaragoza O, Hidalgo-Miranda A, Rodriguez-Dorantes M (2014). MicroRNAs transported by exosomes in body fluids as mediators of intercellular communication in cancer. Onco Targets Ther.

[14] Rayner KJ, Hennessy EJ (2013). Extracellular communication via microRNA: lipid particles have a new message. J Lipid Res.

[15] Kosaka N, Iguchi H, Yoshioka Y, Takeshita F, Matsuki Y, Ochiya T (2010). Secretory mechanisms and intercellular transfer of microRNAs in living cells. J Biol Chem.

[16] Brink PR, Valiunas V, Gordon C, Rosen MR, Cohen IS (2012). Can gap junctions deliver?. Biochim Biophys Acta.

[17] Greco SJ, Rameshwar P (2013). Analysis of the transfer of circulating microRNA between cells mediated by gap junction. Methods Mol Biol.

[18] Suzhi Z, Liang T, Yuexia P, Lucy L, Xiaoting H, Yuan Z, Qin W (2015). Gap Junctions Enhance the Antiproliferative Effect of MicroRNA-124-3p in Glioblastoma Cells. J Cell Physiol.

[19] Wang W, Zhang E, Lin C (2015). MicroRNAs in tumor angiogenesis. Life Sci.

[20] Zhao W, Zhao SP, Zhao YH (2015). MicroRNA-143/-145 in Cardiovascular Diseases. Biomed Res Int.

[21] Chen XJ, Shi KQ, Wang YQ, Song M, Zhou W, Tu HX, Lin Z (2015). Clinical value of integrated-signature miRNAs in colorectal cancer: miRNA expression profiling analysis and experimental validation. Oncotarget.

[22] Matejuk A, Collet G, Nadim M, Grillon C, Kieda C (2013). MicroRNAs and tumor vasculature normalization: impact on anti-tumor immune response. Arch Immunol Ther Exp (Warsz).

[23] Climent M, Quintavalle M, Miragoli M, Chen J, Condorelli G, Elia L (2015). TGFbeta Triggers miR-143/145 Transfer From Smooth Muscle Cells to Endothelial Cells, Thereby Modulating Vessel Stabilization. Circ Res.

[24] Li X, Zhang G, Luo F, Ruan J, Huang D, Feng D, Xiao D, Zeng Z, Chen X, Wu W (2012). Identification of aberrantly expressed miRNAs in rectal cancer. Oncol Rep.

[25] Qin J, Wang F, Jiang H, Xu J, Jiang Y, Wang Z (2015). MicroRNA-145 suppresses cell migration and invasion by targeting paxillin in human colorectal cancer cells. Int J Clin Exp Pathol.

[26] Panza A, Votino C, Gentile A, Valvano MR, Colangelo T, Pancione M, Micale L, Merla G, Andriulli A, Sabatino L, Vinciguerra M, Prattichizzo C, Mazzoccoli G, Colantuoni V, Piepoli A (2014). Peroxisome proliferator-activated receptor gamma-mediated induction of microRNA-145 opposes tumor phenotype in colorectal cancer. Biochim Biophys Acta.

[27] Wang Z, Zhang X, Yang Z, Du H, Wu Z, Gong J, Yan J, Zheng Q (2012). MiR-145 regulates PAK4 via the MAPK pathway and exhibits an antitumor effect in human colon cells. Biochem Biophys Res Commun.

[28] Thuringer D, Berthenet K, Cronier L, Jego G, Solary E, Garrido C (2015). Oncogenic extracellular HSP70 disrupts the gap-junctional coupling between capillary cells. Oncotarget.

[29] Thuringer D, Jego G, Wettstein G, Terrier O, Cronier L, Yousfi N, Hebrard S, Bouchot A, Hazoume A, Joly AL, Gleave M, Rosa-Calatrava M, Solary E, Garrido C (2013). Extracellular HSP27 mediates angiogenesis through Toll-like receptor 3. FASEB J.

[30] Carmeliet P, Jain RK (2000). Angiogenesis in cancer and other diseases. Nature.

[31] Zhang H, Pu J, Qi T, Qi M, Yang C, Li S, Huang K, Zheng L, Tong Q (2014). MicroRNA-145 inhibits the growth, invasion, metastasis and angiogenesis of neuroblastoma cells through targeting hypoxia-inducible factor 2 alpha. Oncogene.

[32] Loewenstein WR (1979). Junctional intercellular communication and the control of growth. Biochimica et biophysica acta.

[33] Budunova IV, Williams GM (1994). Cell culture assays for chemicals with tumor-promoting or tumor-inhibiting activity based on the modulation of intercellular communication. Cell biology and toxicology.

[34] Yamasaki H, Naus CC (1996). Role of connexin genes in growth control. Carcinogenesis.

[35] Paul DL (1986). Molecular cloning of cDNA for rat liver gap junction protein. The Journal of cell biology.

[36] Lee SK, Teng Y, Wong HK, Ng TK, Huang L, Lei P, Choy KW, Liu Y, Zhang M, Lam DS, Yam GH, Pang CP (2011). MicroRNA-145 regulates human corneal epithelial differentiation. PLoS One.

[37] Klotz LO (2012). Posttranscriptional regulation of connexin-43 expression. Arch Biochem Biophys.

[38] Kandouz M, Batist G (2010). Gap junctions and connexins as therapeutic targets in cancer. Expert Opin Ther Targets.

[39] Anderson C, Catoe H, Werner R (2006). MIR-206 regulates connexin43 expression during skeletal muscle development. Nucleic Acids Res.

[40] Osbourne A, Calway T, Broman M, McSharry S, Earley J, Kim GH (2014). Downregulation of connexin43 by microRNA-130a in cardiomyocytes results in cardiac arrhythmias. J Mol Cell Cardiol.

[41] Jin Z, Xu S, Yu H, Yang B, Zhao H, Zhao G (2013). miR-125b inhibits Connexin43 and promotes glioma growth. Cell Mol Neurobiol.

[42] Hao J, Zhang C, Zhang A, Wang K, Jia Z, Wang G, Han L, Kang C, Pu P (2012). miR-221/222 is the regulator of Cx43 expression in human glioblastoma cells. Oncol Rep.

[43] Mothersill C, Seymour CB (2004). Radiation-induced bystander effects--implications for cancer. Nat Rev Cancer.

[44] Carystinos GD, Katabi MM, Laird DW, Galipeau J, Chan H, Alaoui-Jamali MA, Batist G (1999). Cyclic-AMP induction of gap junctional intercellular communication increases bystander effect in suicide gene therapy. Clin Cancer Res.

