# Correction: Gap junction-mediated transfer of miR-145-5p from microvascular endothelial cells to colon cancer cells inhibits angiogenesis

**DOI:** 10.18632/oncotarget.28528

**Published:** 2024-05-07

**Authors:** Dominique Thuringer, Gaetan Jego, Kevin Berthenet, Arlette Hammann, Eric Solary, Carmen Garrido

**Affiliations:** ^1^INSERM, U866, Faculty of Medecine, 21000 Dijon, France; ^2^University of Bourgogne-Franche-Comté, 21000 Dijon, France; ^3^INSERM, U1170, Institut Gustave Roussy, 94508 Villejuif, France; ^4^CGFL, BP77980, 21000 Dijon, France

**This article has been corrected:** Because a previously published image was used in Figure 1E, the authors have updated the figure legend as shown below. The authors declare that these corrections do not change the results or conclusions of this paper.

Original article: Oncotarget. 2016; 7:28160–28168. 28160-28168. https://doi.org/10.18632/oncotarget.8583

**Figure 1:**
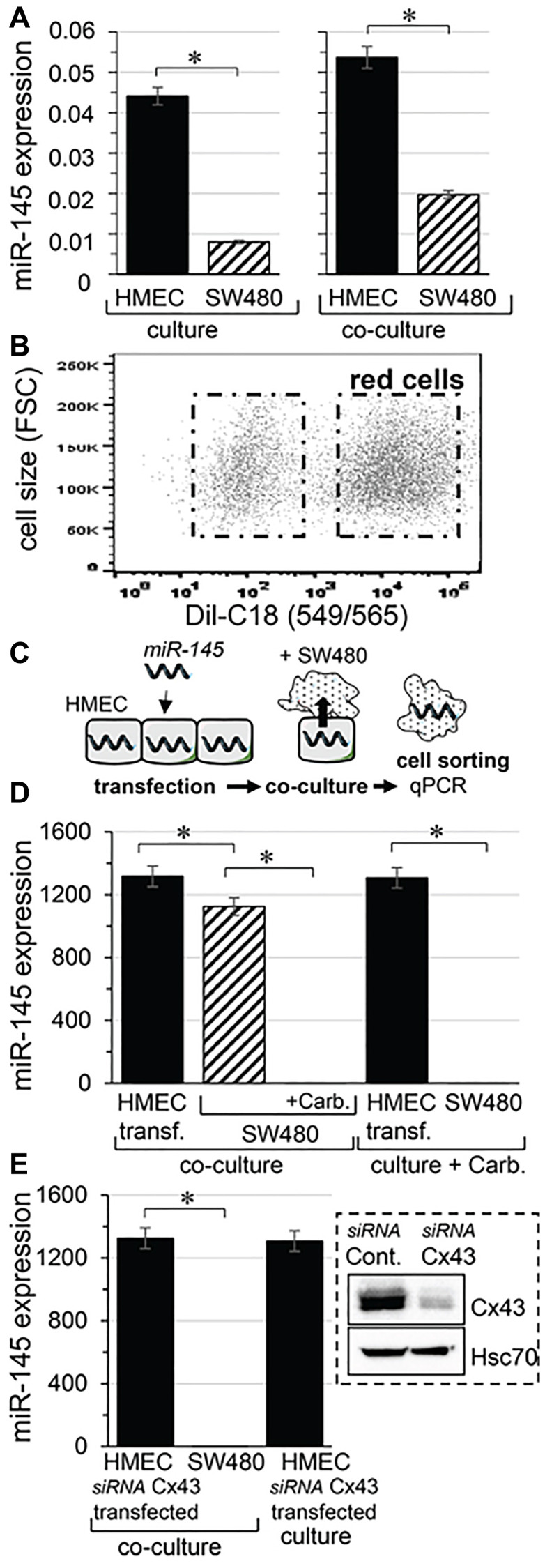
Micro-RNA transfer from microvascular endothelium (HMEC) to colorectal cancer cells (SW480). (**A**) Expression profile of miR-145 in HMEC (black) and SW480 (hatched), separately cultured (left) or co-cultured (right) for 12 hours. Levels of miR-145 were expressed relative to levels of U6 snRNA, commonly used as an internal control in miRs analysis (means ± SD; ^*^ *P* < 0.05; *n* = 3). (**B**) Cell sorting by flow cytometry. SW480 were labelled with the fluorescent dye DiL-C18 (red cells), then plated with unlabeled HMEC in a ratio of 1:1. (**C**) Scheme illustrating the procedure to determine the transfer of microRNA (miR-145) through hetero-cellular gap junction channels established between HMEC and SW480. (**D**) Transfer of miR-145 to SW480 is inhibited by a gap junction blocker. HMEC loaded with miR-145-5p mimic (30 nM) were co-cultured with SW480, in the presence or the absence of carbenoxolone (carb. 100 µM). The miR-145 levels measured in donor HMEC (black) and receiver SW480 (hatched), after 12 h of co-culture. Note that carbenoxolone does not affect the miR-145 expression in transfected HMEC or in cancer cells cultured separately. (**E**) Down-regulation of Cx43 expression in HMEC does not affect loading of miR-145 mimic but suppresses transfer of miR-145 to SW480. Right insert, representative immune-blot of Cx43 protein level in HMEC transfected with control siRNA or siRNA Cx43 ([28]; Hsc70 as loading control). Note that the siRNA Cx43 transfection of HMEC does not affect their loading with miR-145-5p mimic. D, E. Values of miR-145-5p expression relative to U6 snRNA in each cell type and condition, are means ± SD of triplicate measurements from three experiments; ^*^ *P* < 0.5 vs. donors (Mann-Whitney *U* test and Kruskal-Wallis test; *n* = 3).

## References

[28] Thuringer D , Berthenet K , Cronier L , Jego G , Solary E , Garrido C . Oncogenic extracellular HSP70 disrupts the gap-junctional coupling between capillary cells. Oncotarget. 2015; 6:10267–83. 10.18632/oncotarget.3522. 25868858 PMC4496354

