# Supplementary material for: Gap junction-mediated transfer of miR-145-5p from microvascular endothelial cells to colon cancer cells inhibits angiogenesis
Source: Oncotarget. 2016 Apr 5;7(19):28160–8. doi: 10.18632/oncotarget.8583 (PMC5053717; doi:10.18632/oncotarget.8583)
Supplement: Supplementary file 1 [file oncotarget-07-28160-s001.pdf]

SUPPLEMENTARY FIGURE

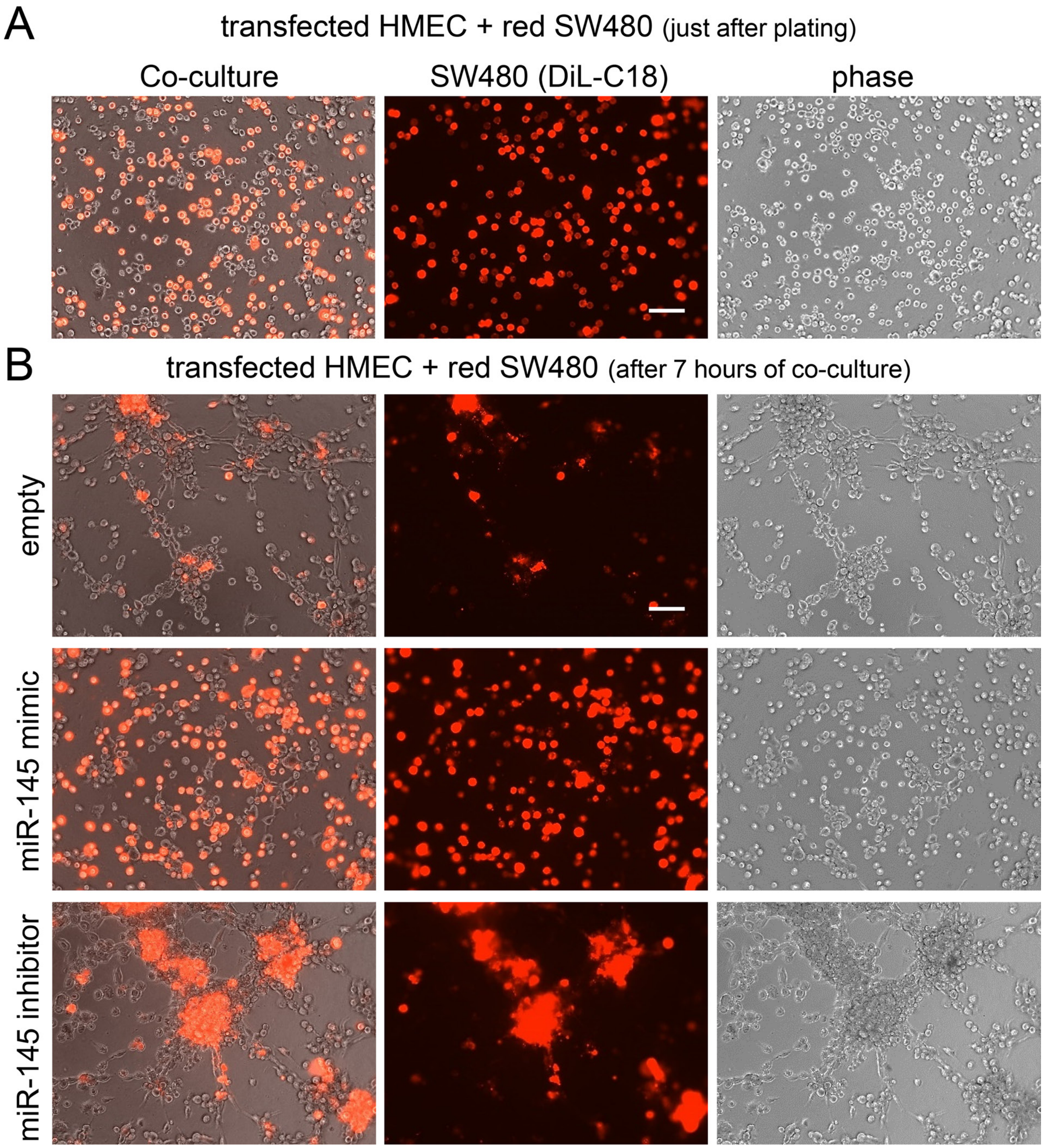

Supplementary Figure S1: miR-145-5p inhibits angiogenic effects of cancer cells. *In vitro* tubulogenesis assay of HMEC loaded or not (empty) with miR-145-5p mimic or inhibitor (60 nM). HMEC were plated with red SW480 cells (labelled with DiL-C18), and photographed: **A.** just after plating (ratio 1:1); **B.** after 7 hours of co-culture. Note the adhesion of cancer cells to endothelial cells (Bar 80 µm; representative of 3 independent experiments).
